# Immobilisation of Delta-like 1 ligand for the scalable and controlled manufacture of hematopoietic progenitor cells in a stirred bioreactor

**DOI:** 10.1186/s12896-017-0383-0

**Published:** 2017-08-04

**Authors:** Rebecca L. L. Moore, Matthew J. Worrallo, Peter D. Mitchell, Jon Harriman, Katie E. Glen, Robert J. Thomas

**Affiliations:** Healthcare Engineering Research Group, Centre for Biological Engineering, Wolfson School of Mechanical and Manufacturing Engineering, Loughborough University, Loughborough, Leicestershire UK

**Keywords:** Cell culture, Ex vivo expansion, Delta-like 1, Hematopoietic progenitor cell, Immobilised, Manufacturing, Notch, Scalable

## Abstract

**Background:**

Umbilical cord blood provides a source of hematopoietic stem cells for transplantation with immunological and availability advantages over conventional bone marrow sources. Limited cell numbers and slower engraftment from umbilical cord blood units has led to the clinical development of immobilised Notch ligand Delta-Like 1 to promote ex vivo expansion of a rapidly engrafting cell population. However, current immobilisation methods are not simple to scale in a controlled manner.

**Results:**

Delta-Like 1 was immobilised onto streptavidin coated magnetic particles via a heterobifunctionalised polyethylene glycol linker molecule to provide an easily manipulated format of surface protein presentation. CD34^+^ enriched cord blood cells were treated with Delta-Like 1 immobilised particles, and immunophenotypic markers measured to monitor population distributions using cluster identification, characterization, and regression software. The amenability of the approach to scalability was evaluated in a micro-scale stirred tank bioreactor. Surface concentration of Delta-Like 1 was well controlled used differing stoichiometric reagent ratios. Protein immobilisation was a cost effective process and particles were efficiently removed from the final cell product. Immobilised Delta-Like 1 is functional and stimulates qualitatively similar CD34^hi^, CD38^lo^, CD90^lo^, CD133^hi^, CD135^hi^ progenitor expansion in both static culture and scalable stirred culture platforms.

**Conclusions:**

Immobilised Delta-Like 1 in this form has the potential to improve the manufacturing efficiency and control of final ex vivo expanded cell product through compatibility with highly controlled and characterised suspension culture systems.

**Electronic supplementary material:**

The online version of this article (doi:10.1186/s12896-017-0383-0) contains supplementary material, which is available to authorized users.

## Background

Hematopoietic stem cell (HSC) transplantation is an effective therapy for numerous hematopoietic disorders. HSCs are available from various human sources: mobilised peripheral blood (MPB), bone marrow (BM), and umbilical cord blood (UCB). Although all have shown clinical efficacy, HSCs derived from UCB eliminates risk to the donor, are readily available as a sustainable source, offer a lower transmission rate of infectious and genetic diseases, and are more tolerant of immunological mismatches compared to those from other sources [1]. The immunological properties significantly improve the likelihood of donor matching and reduce the incidence of graft versus host disease (GVHD) [2]. Successful transplantation relies upon the function of long and short term repopulating HSCs, as well as a short time to engraftment into the BM to minimise post-transplant susceptibility to infection and bleeding disorders. One of the key drawbacks of UCB as a source is the inability to obtain sufficient numbers of HSCs from a single cord for transplantation into the average adult patient; this contributes to prolonged phases of neutropenia, thrombocytopenia and suboptimal engraftment into the recipient BM [3–7].

The ex vivo expansion of UCB cells is one way to overcome the limited cell numbers available. Upon expansion HSCs are capable of either self-renewal or differentiation to lineage committed cells; an appropriate balance in this expansion phase is required to produce a cell population with the necessary engraftment characteristics. Supportive conditions and factors for primitive cell expansion have been identified, including the key cytokines stem cell factor (SCF), flt3/flk2 ligand (FLT), and thrombopoietin (TPO) [8–11]. However, such cytokine mediated expansion methodologies have not shown significant clinical improvement in time to engraftment [12–14]. Co-culture with other cell types is another approach being investigated by researchers to improve expansion and lower the time to engraftment with good success [15], however this method relies upon the introduction of a contaminating cell type and presents more challenges in downstream processing [16]. Factors which generate a population that retains long term repopulating ability and also boosts the progenitor cells responsible for rapid engraftment, through various intrinsic signalling pathways, are being investigated clinically [13, 17, 18]. One candidate, the Notch pathway, is well documented to have a modulatory effect on the differentiation of stem cell systems including various effects on HSC and hematopoietic progenitor proliferation and commitment [19, 20]. Notch signalling is mediated by interactions between transmembrane receptors, including members of the Delta like (DL1, DL3, DL4) family and their membrane bound ligand. Ex vivo expansion of HSC progenitors in the presence of immobilised DL1 (iDL1) produces an approximate 100-fold greater increase of CD34^+^ cells relative to a non-DL1 control [21]. These cells have enhanced repopulating ability in a sub-lethally irradiated mouse model, notably substantially faster and higher levels of myeloid and lymphoid engraftment [22].

A phase 1 clinical trial to identify whether CD34^+^ enriched UCB could be expanded in the presence of immobilised DL1 to accelerate hematopoietic recovery recently met its primary objective and was deemed safe [23]. The culture process utilised X-fold™ tissue culture bags and Nunc flasks [24]. Infused CD34^+^ number has been identified as one of the best indicators for rapid hematopoietic recovery [25]. Due to feedback from mature cells influencing lineage development during the expansion process [26], culture densities are kept relatively low, typically below 5 × 10^5^ mL^−1^. In order to produce the CD34^+^ cell numbers (2-5 × 10^6^) reported large medium volumes are required [27]. Furthermore, a non-mixed culture flask does not provide a scalable and homogenous interaction between surface presented ligand and cells. Given the reported dose-dependence of engraftment on DL-1 exposure, such controllable interaction would be necessary for process reproducibility across scales [21].

Herein we describe the controlled immobilisation of biotinylated DL-1 onto streptavidin coated magnetic particles to enable incorporation into a highly controlled and volume scalable suspension culture system. The magnetic nature allows for the removal of the particles from ex vivo culture vessels at specific time points, independent of other medium components, enabling particle recycling through medium exchange. Further, DL1 remained functional upon immobilisation and during culture agitation, and exhibited a controllable response via pronounced retention of progenitor cells in culture.

## Methods

### iDL1 particle preparation

iDL1 particles were produced using one of two routes described as forward or reverse. Forward: lyophilised carrier free DL1 (R and D systems, Abingdon, UK) was suspended at 1 μM (unless otherwise stated) in either sterile PBS or anhydrous DMSO. Biotin polyethylene glycol (PEG) N-Hydroxysuccinimide (NHS), MW 2000, (NANOCS, New York, USA) was suspended in anhydrous DMSO at the required concentration for the individual experiment being carried out. Equal volumes of DL1 and heterobifunctionalised PEG molecule were homogenised in an Eppendorf LoBind microcentrifuge tube and incubated at room temperature for 2 h (unless otherwise stated). MagnaLink Steptavidin Magnetic Beads (Nominal mean bead diameter: 3 μm, Solulink, San Diego, USA) were prepared by thoroughly mixing the stock solution, aliquoting the required particle number into a LoBind microcentrifuge tube and spinning the particle suspension at 300 g for 8 min before removing the supernatant. Biotinylated DL1 conjugates were supplemented to the prepared 2.8 μm streptavidin coated magnetic particles and incubated overnight at 4–8 °C with constant mixing. Particles were washed 5 times in 1 mL of PBS before DL1 quantification using flow cytometry. Reverse: heterobifunctionalised PEG molecules of various molecular weights, (2000, 5000 and 10,000) were suspended in anhydrous DMSO at a concentration of 1 mM. Prepared MagnaLink Steptavidin Magnetic Beads were added to the heterobifunctionalised PEG molecule solution at approximately 2 × 10^5^ particles μL^−1^ and incubated for 16 h unless otherwise stated. Particles were washed 5 times in 1 mL of anhydrous DMSO using a magnetic plate rack (BioRad, California, USA) in order to remove unbound heterobifunctionalised PEG molecules. Lyophilised carrier free DL1 was suspended at 1 μM, unless otherwise stated, in either sterile PBS or anhydrous DMSO and added directly to dry biotinylated particles. Particles were incubated for 2 h, unless otherwise stated, at room temperature with constant mixing. After incubation particles were washed 5 times in 1 mL of PBS before DL1 quantification using flow cytometry.

### Quantification of DL1 immobilisation

2 × 10^5^ magnetic particles were stained for 30 min at room temperature with 5 μL of PE Mouse Anti-Human Delta-Like Protein 1 (BD Biosciences, Oxford, UK) in 50 μL of stain buffer (ThermoFisher, London, UK). As a negative control 2 × 10^5^ magnetic particles were stained in parallel with a PE IgG_1_ isotype control. After incubation particles were washed once with PBS before being resuspended in 100 μL of stain buffer for analysis. Florescent reference standards (Quantum MESF; Bangs Labs Inc., Indianapolis, USA) were used to determine the mean mass of DL1 per particle using the net geometric mean of fluorescent intensity.

### Cell culture

Cells were obtained as research units from the Anthony Nolan Cell Therapy Centre (ANCTC). ANCTC obtain tissue with informed consent and supply for specific projects approved by their board under their generic research tissue bank ethics. CD34^+^ enriched umbilical cord-derived cells were obtained from 20 pooled UCB mononuclear cells (MNCs) via positive selection using CD34 MicroParticle Kit (MACS; Miltenyi Biotec, Bergisch Gladbach, Germany). The CD34^+^ percentage of enriched cells ranged from 92% to 97% post thaw as assessed by flow cytometry. Isolated cells were cryopreserved prior to experiments. Full phenotypic characterisation post thaw can be found in Additional file 1. Cells were cultured in Iscove’s modified Dulbecco’s medium with GlutaMAX-I (Invitrogen, Paisley, UK) supplemented with 20% (*v*/v) BIT 9500 Serum Substitute (Stemcell Technologies, Vancouver, Canada), 2% (*v*/v) GlutaMAX (ThermoFisher) and cytokines known to support HSC expansion, 100 ngmL^−1^ Stem Cell Factor (SCF), Fms Related Tyrosine Kinase 3 (FLT3) and 50 ngmL^−1^ Thrombopoietin (TPO) (R and D Systems) [26]. Cells were seeded at 1 × 10^5^ mL^−1^ and were reset back to initial seeding density every 3 days through centrifugation and re-suspension of cells in a 1:1 replacement of spent medium:fresh medium, cytokines were added at 2× concentration in fresh medium to replace degraded cytokines in the spent fraction. iDL1 particles were added at time 0 h and retained in each treated sample during medium exchange using a magnet. To control for any effects of soluble DL1 or blank particles three different controls were included: untreated, soluble DL1 and blank particles. Cells within the untreated control group were expanded devoid of DL1 or particles. Cells within the soluble DL1 control group were treated with 50 ng/mL of DL1, equivalent to the highest concentration present in the treatment group. Cells with in the blank particle group were treated with particles exclusive of DL1, again at equivalent levels with the highest concentration present in the treatment group. For static conditions the number of biological replicates totalled five, (*n* = 5) for the bioreactor conditions three biological replicates were performed (*n* = 3). Expansion data was interrogated for outliers using a Grubbs’ test. Outliers were not included in the data analysis.

### Bioreactor setup and maintenance

The ambr® bioreactor (TAP Biosystems, Royston UK) is a scaled-down version of a classic stirred tank bioreactor operating at micro-scale (10–15 mL), using multiple disposable micro-bioreactors, with individual, automated, online monitoring and control of pH, oxygen gassing, temperature and stir rate. The system has been validated for 2 L scale-up equivalence [28]. Before cell defrost, ambr® vessels were loaded with 14 mL of medium and stabilised for temperature (37 °C), oxygen delivery (atmospheric) and pH (7.4). Culture pH was maintained by automated sodium bicarbonate (20 μL of 1 molL^−1^ solution: Sigma-Aldrich) additions at 2 h intervals (if required) and regulated carbon dioxide gassing. Impeller speed was 450 rpm, experimentally determined as the slowest speed to keep particles in a homogenous suspension [29, 30].

### Cell phenotyping

The antigenic phenotype of UCB cells was assessed before and during ex vivo expansion. Approximately 1 × 10^5^ cells per test were incubated with the required antibody panel as per manufacturer’s instructions for 30 min at room temperature. All antibodies were preconjugated and monoclonal. Progenitor panel: CD34-PerCP-Vio700, CD38-FITC, CD45RA-VioGreen, CD90-APC-Vio770, CD133-APC, CD135-PE-Vio770 (Miltenyi Biotech) and CD33-PE (BD Biosciences). Mature stage myeloid panel: CD13-APC, CD14-PE, CD15-VioGreen, CD34-PerCP-Vio700, CD33-APC-Vio770, CD38-FITC, CD123-VioBlue (Miltenyi Biotech). Analysis was conducted with CITRUS (cluster identification, characterisation and regression) software [31]. The software uses a regularized supervised learning algorithm to determine the populations and features of the samples being analysed in a correlative manner. The results of a CITRUS run are clusters (populations) that differentiate the observed endpoint of the samples, and the features (relative population abundance or median expression of a functional marker) of the clusters that are responsible. All CITRUS analyses were conducted using a Significance Analysis of Microarrays (SAM) association model and a minimum cluster size of 4%, at least 5000 events from each sample were included for the analysis.

### Statistics

One-way ANOVA (analysis of variance) with post-hoc Tukey HSD (honestly significant difference) test calculator for comparing multiple treatments was used to conduct statistical analysis. All values are reported as the mean ± SD. Values were considered statistical significant for *P* < 0.05.

## Results

### Optimal conditions for the immobilisation of Delta-like 1

**Fig. 1:**
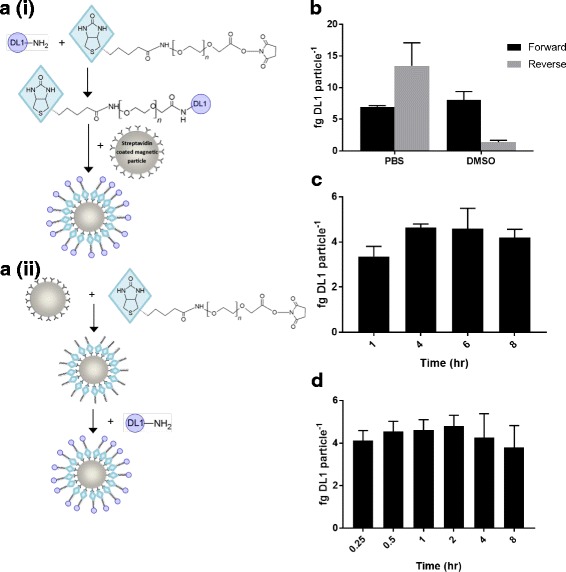
Delta-Like 1 can be reliably biotinylated and immobilised onto streptavidin coated magnetic micro particles. DL1 can be immobilised using two distinct reaction pathways, **a(i)** forward, and **a(ii)** reverse. In the forward pathway DL1 primary amines first react with the NHS functionality of the heterobifunctionalised PEG molecule. Once reacted, streptavidin coated magnetic particles are added allowing the biotin functionality to bind with the streptavidin, resulting in iDL1. In the reverse pathway the heterobifunctionalised PEG molecule first reacts with the streptavidin coated particle, the DL1 is then added and reacts with the NHS functionality to produce iDL1 (*n* = 3). **b** DL1 could be immobilised via both pathways, the forward pathway demonstrated no effect of the DL1 solvent. The reverse pathway immobilised most DL1 per particle when DL1 was dissolved in PBS (*n* = 3). **c** Optimisation of the reverse pathway for maximum DL1 immobilisation demonstrated a rapid saturation of binding between streptavidin and the heterobifunctionalised PEG molecule during the first stage of the synthesis (*n* = 3) and **d** a 2 h optimal incubation time for the second stage (*n* = 3)

The synthesis of an immobilised ligand complex using streptavidin/biotin interactions can take place via two discreet pathways: attaching the heterobifunctionalised PEG molecule to the protein followed by incubation with the particle (forward), or attaching the heterobifunctionalised PEG molecule to the streptavidin particle followed by incubation with the protein (reverse) (Fig. 1a). Further, solvent choice can heavily influence the dissolution and reaction of many protein reagents [32]. In order to identify the conditions which led to maximum DL1 immobilisation the sequence of reaction steps and the solvent, PBS or DMSO, in which the protein was dissolved, were evaluated. It was observed that DL1 was immobilised at highest levels when dissolved in PBS and processed via the reverse reaction pathway (Fig. 1b). However, the solvent and reaction sequence were non-independent; the outcome of the reverse reaction was highly sensitive to solvent whereas the forward reaction was not. This is likely to be due to the different reaction sequences undergoing differing levels of hydrolytic degradation of the NHS group and/or protein denaturing in DMSO. The previously identified most effective immobilisation pathway (reverse/PBS pathway) was evaluated at each step for effect of reaction times upon protein immobilisation. Maximal binding for the first stage of the reaction, between the streptavidin coated particle and heterobifunctionalised PEG molecule, occurred rapidly with no additional binding observed after 4 h (Fig. 1c). The second stage of the process, the reaction between the NHS group of the heterobifunctionalised PEG molecule and primary amines present in DL1, was also rapid with no significant increase in binding observed after 30 min incubation (Fig. 1d).

### Factors controlling surface concentration of Delta-like 1

**Fig. 2:**
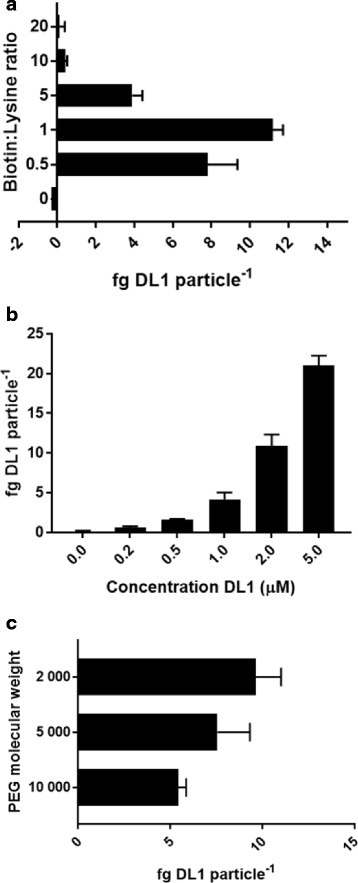
Surface concentration of immobilised Delta-Like 1 can be controlled during particle synthesis. **a** Using the forward reaction pathway it is possible to control the presentation of DL1 by regulating the ratio of the heterobifunctionalised PEG molecule to the number of primary amine groups present in DL1 (*n* = 3). **b** Using the reverse synthetic pathway, DL1 immobilisation increases with increasing DL1 concentration in the second stage of the process (*n* = 3). **c** The use of increased PEG chain lengths within the heterofunctionalised biotin molecule results in a reduction in DL1 immobilisation using the reverse synthetic method (*n* = 3)

It is known that certain proteins require particular spatio-temporal presentation with respect to their receptors to elicit specific cellular responses [33]. Planar surface iDL1 concentration can be varied in order to quantitatively regulate the induction of Notch signalling which in turn differentially affects HSC lineage commitment [22]. We therefore explored methodological control of the concentration of iDL1 presented on the particle surface. Although the reverse pathway was favourable for maximum DL1 immobilisation, both forward and reverse have the potential to control DL1 surface concentration. In the forward pathway increasing the concentration of the heterobifunctionalised PEG molecule in excess of DL1 lysine residues would be anticipated to lead to unreacted NHS moieties. Heterobifunctionalised PEG molecules not bound to DL1 would subsequently reduce binding of protein to the particles via the competitive blanking of streptavidin. In the reverse case, increasing the DL1 concentration during synthesis would favour reaction of NHS with protein relative to hydrolytic degradation and therefore reduce the level of non-protein bound biotin-PEG available to blank the particles. In accordance with this, the molar ratio of heterobifunctionalised PEG molecule:DL1 primary amine in the forward synthetic pathway significantly influenced the concentration of iDL1; binding was highest when the heterobifunctionalised PEG molecule concentration was matched to the binding sites in the protein and fell sharply either side (Fig. 2a). Using the reverse pathway it was possible to alter the amount of iDL1 by simply altering the concentration of DL1 in the second step of the reaction pathway (Fig. 2b). The final factor of control investigated was the PEG length within the heterobifunctionalised PEG molecule. It was observed that an increase in PEG length resulted in a reduction of iDL1, presumably due to stearic hindrance (Fig. 2c).

### Investigating manufacturing challenges of the use of immobilised Delta-like 1

**Fig. 3:**
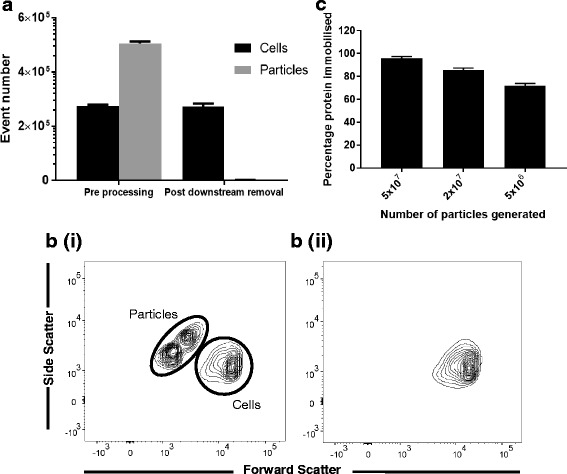
Investigating manufacturing challenges of the use of immobilised Delta-Like 1. Efficacy of the immobilisation process was evaluated using an ELISA immunoassay. **a** Efficacy was dependent on the number of particles generated, with up to 96% of the protein immobilised (*n* = 3). Removal of magnetic particles was evaluated by flow cytometry. **b** Voltage setting and gating was optimised to assess particle number in culture (i) and after removal from culture (ii), **c** removal of magnetic particles from cells at the end of the culture period was evaluated, MACS^®^ columns removed 99.6% of particles with minimal cell loss (<1%) (*n* = 6)

For immobilised proteins to be considered as an alternative to soluble the immobilisation process must be cost effective and the particles efficiently removed from the cell product in downstream processing. To determine the level of protein utilisation during the process, DL1 concentration in the supernatant was measured before and after immobilisation. Protein immobilisation was found to be highly efficient at defined particle:protein concentration ratios (Fig. 3a), with up to 96% of the protein immobilised. It is also important for ancillary materials to be easily and reliably removable from any cell therapy product to aid with regulatory approval [16]. Further, efficient removal will enable simple exploration of alternative temporal exposure to DL1 in future studies, and process control, independent of other media elements. We therefore explored the conditions required to remove the magnetic particles from HSC culture. MACS® columns were utilised to successfully remove magnetic particles from the cell product at the end of the culture period. Magnetic particle levels were reduced to 0.40% of the original concentration, as measured by flow cytometry, with minimal cell loss (<1%) and no impact on cell viability (Fig. 3b).

### Functionality of immobilised Delta-like 1 in static culture

**Fig. 4:**
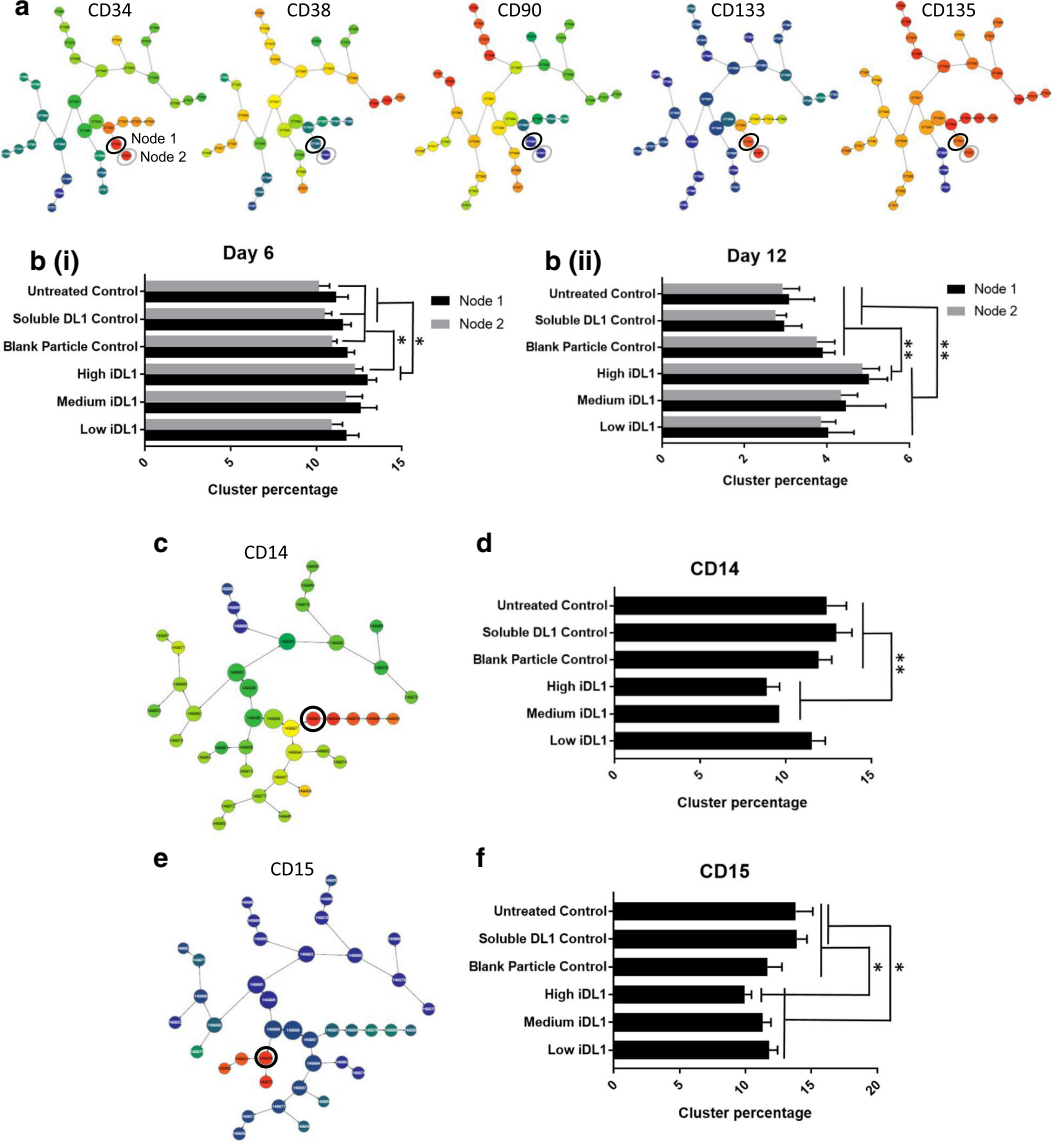
Immobilised Delta-Like 1 can induce skewed lineage output of hematopoietic progenitor cells in static culture. CD34^+^ enriched cells (95% CD34^+^) were cultured with iDL1 at three concentrations alongside three controls: untreated, soluble DL1 and blank particles only (*n* = 5). **a** FCS files were uploaded into Cytobank and CITRUS cluster trees derived, clustering on CD34, CD33, CD38, CD45ra, CD90, CD133 and CD135 marker intensities. Cluster trees of CD34, CD38, CD90, CD133 and CD135 are shown, identifying the progenitor nodes defined phenotypically as CD34^hi^CD38^neg^CD90^neg^CD133^hi^CD135^hi^. **b** Progenitor nodes (identified in 4A) were evaluated for their percentage abundance at day 6 (i) and day 12 (ii), cells cultured with high levels of iDL1 contained a significantly higher percentage of progenitor cells compared to all controls. All concentrations of iDL1 significantly improved progenitor abundance compared with untreated and soluble DL1 controls at day 12. Further phenotypic analysis was undertaken at day 12 to evaluate the expression of more mature surface markers. FCS files were uploaded into Cytobank and CITRUS cluster trees derived, clustering on CD13, CD14, CD15, CD24, CD33, CD38 and CD123 marker intensities. **c** Cluster tree of CD14 expression, the parent CD14^hi^ node was identified. **d** Evaluation of percentage abundance within the parent CD14^hi^ node at day 12 identified culture with high and medium levels of iDL1 resulted in significantly less mature CD14^hi^ cells compared to all controls. **e** Cluster tree of CD15 expression, the parent CD15^hi^ node was identified. (F) Evaluation of percentage abundance within the parent CD15^hi^ node at day 12 identified culture with high levels of iDL1 resulted in significantly less mature CD15^hi^ cells compared to all controls. All concentrations of iDL1 significantly decreased CD15^hi^ abundance compared with untreated and soluble DL1 controls. Values were considered (*) statistically significant for *P* < 0.05, (**) very statistically significant for *P* < 0.01

iDL1 particles were added to UCB derived CD34^+^ enriched HSC culture in order to evaluate whether the immobilised ligand complexes retained functionality to retain progenitor cells during expansion. Particles and cells remained in single suspension, with less than 0.1% of cells forming aggregates as assessed by eye at 20× and 40× magnification (Additional file 2). Cell viability was not impacted by treatment type, with cell viability remaining above 97% throughout the culture period. The fold expansion of the viable cell population ranged between 310 and 563, the bead only control showed a significant increase in expansion over the other treatment types (*p* < 0.05). HSC progenitor and myeloid lineage was assessed using flow cytometry to measure the cell surface antigen expression of CD33, CD34, CD38, CD45RA, CD90, CD133, CD135, CD13, CD14, CD15, CD123. A computational approach, cluster identification, characterisation and regression (CITRUS), was applied to analyse flow cytometry data. CITRUS overcomes a number of limitations of conventional approaches to analysis and visualisation of multi-dimensional flow data such as subjective user input, labour intensity and requirement for prior knowledge or assumptions of the biological system [31]. CITRUS organises cells into statistically distinct populations based on clustering of marker expression and generates a 2D phenotypic tree diagram based on phenotypic proximity of populations. Analysis of data via this methodology results in a cluster tree for each of the clustering markers, five of the clustering markers which contribute to progenitor analysis are shown (Fig. 4a). Evaluation at day 6 and day 12 of the culture period showed consistent bias in the percentage of cells within progenitor clusters, denoted by CD34^hi^CD38^neg^CD90^neg^CD133^hi^CD135^hi^ (Fig. 4b, c) [34–37]. Cells treated with high levels of iDL1 had significantly higher levels of progenitor cells at both day 6 and 12 when compared to the three control conditions. It was further observed that at the end of the culture period (day 12) cells treated with all three levels of iDL1 had significantly more cells in the progenitor nodes than the untreated and soluble DL1 controls in a concentration dependant manner. Further analysis on day 12 of more mature surface antigens [21, 22] identified a significant decrease in CD14^hi^ (Fig. 4d, e) and CD15^hi^ (Fig. 4f, g) cells when treated with iDL1 compared with control treatments again in a dose dependent manner. The dose dependent nature of the effect alongside the consistent higher progenitor cell phenotype with decreased mature antigen expression when cells are treated with iDL1 demonstrates the ability of particle presented DL1 to retain progenitor cell output.

### Functionality of immobilised Delta-like 1 in stirred bioreactor

**Fig. 5:**
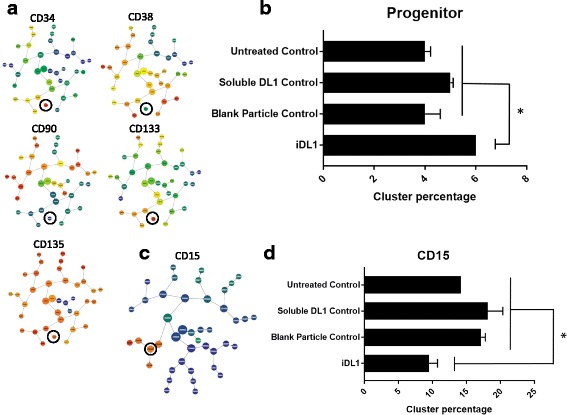
Immobilised Delta-Like 1 induces the same lineage skewing of hematopoietic progenitor cells in stirred bioreactor systems. CD34^+^ enriched cells (95% CD34^+^) were cultured with iDL1, alongside untreated, soluble DL1 and blank particles as controls, in a mechanically agitated suspension bioreactor (*n* = 3). **a** As previously shown in the static platform, FCS files were uploaded into Cytobank and CITRUS cluster trees derived, clustering on CD34, CD33, CD38, CD45ra, CD90, CD133 and CD135 marker intensities. Cluster trees of CD34, CD38, CD90, CD133 and CD135 are shown, identifying the progenitor node defined phenotypically as CD34^hi^CD38^neg^CD90^neg^CD133^hi^CD135^hi^. **b** The addition of iDL1 to CD34^+^ enriched cells in a stirred culture system led to the same trend in lineage skewing previously identified in static. Evaluation of the percentage abundance within the progenitor node at day 12 identified high levels of iDL1 resulted in significantly more progenitor cells compared to all controls. **c** Cluster tree of CD15 expression, the parent CD15^hi^ node was identified. **d** Evaluation of percentage abundance within the parent CD15^hi^ node at day 12 identified, as in the static platform, culture with high levels of iDL1 resulted in significantly less mature CD15^hi^ cells compared to all controls. Values were considered (*) statistically significant for *P* < 0.05

Demonstration that iDL1 could similarly skew the lineage output of CD34^+^ enriched cells in favour of an earlier progenitor population in a stirred bioreactor was important to show potential for scalable and controlled application. CD34^+^ enriched cells cultured with iDL1 in the stirred reactor again showed no proliferative disadvantage relative to the three controls. Again cell viability was not impacted by treatment type, with cell viability remaining above 95% throughout the culture period. Total cell fold expansion showed no significant difference between treatment groups (range 142–177 fold). Cells were analysed at the end of the culture period using the same CITRUS analysis (Fig. 5a). As observed in static culture, cells treated with iDL1 contained significantly more progenitor cells than the three controls. Further analysis of more mature surface antigens showed no effect on CD14 but did again significantly decrease the level of cells expressing CD15^hi^ (Fig. 5c, d). Finally, in order to test the longevity of iDL1 particles in a stirred culture platform, particles were quantified daily for their DL1 surface concentration throughout the culture period. A fitted exponential decay model gave a half-life of approximately 490 h (R^2^ = 67%).

## Discussion

The role of Notch signalling in the expansion of HSCs, and the potential for therapeutic application, has been widely reported. Notch mediated expansion of UCB progenitor cells results in significant proliferation of precursor cells capable of rapid multi-lineage in vivo NOD/SCID reconstitution [22]. However, efficient Notch signalling in HSCs requires immobilisation of a Notch ligand DL1 and in current protocols this immobilisation is achieved on planar cell culture plastic [38]. The requirement for surface presented ligand complicates simple application of suspension culture systems and development of efficient and predictably scalable methods. Micro-particles are an ideal carrier for immobilised ligands as they offer a large surface area to volume ratio and allow for control of localised concentration and total culture concentration independently. Furthermore they allow for the ligands to be removed from the final cell product, removing the risk of inadvertent activation of undesired signalling pathways in the recipient. They also have the benefit of being compatible with suspension manufacturing systems such as stirred tank bioreactors, and scalable in such systems with a consistent cell surface area to cell interaction. Compatibility with well mixed stirred systems offers a process developer a host of additional optimisation and control opportunities such as precise gas, pH and mechanical stress control.

Herein we have described the immobilisation of Notch ligand DL1 onto the surface of magnetic micro particles. DL1 surface concentration can be controlled via two alternate methods, remains functional under stirred conditions, and can be efficiently withdrawn from culture using simple magnetic methods. In particular, the scale of change of immobilised DL1 concentration achieved via the reverse reaction in response to the change in DL1 reaction concentration suggests this method would offer reproducible control of immobilisation at the fg/bead level. Further, the kinetics underlying this reaction (different sensitivity of rate of hydrolysis of NHS to temperature compared with NHS-protein reaction) suggest efficiency of protein immobilisation should be further tuneable by reaction temperature [39] i.e. colder reaction temperatures would likely require lower protein concentration for equivalent iDL1, reducing manufacturing costs. The immobilisation process was demonstrated to be highly economical with 96% of the input protein immobilised. The efficiency of particle removal must be considered during downstream processing, as a high loss in cell yield would impact the feasibility of such approach. We demonstrated the particles can be effectively removed at the end of the expansion period with minimal cell loss and no impact of cell viability.

Physical forces can influence how receptors transduce signals [40]. The generally observed lack of functionality of Notch ligands in soluble form is hypothesised to be due to a mechanotransduction model of receptor activation [41]. Immobilised DL1 particles were functional. Cells treated with high levels of iDL1 contained a significantly higher percentage of progenitor cells compared to the three controls in both static and stirred culture platforms. Additionally iDL1 performed in a concentration dependent fashion on progenitor retention and the suppression of cells expressing more mature surface antigens.

Laboratory to laboratory comparisons of phenotypic changes measured by flow cytometry are challenging due to subjectivity in analysis [42]. The use of CITRUS software to identify statistically distinct populations based on cell expression of two different 7 antigen marker panels, progenitor and myeloid, removed any subjectivity in cell gating and did not necessitate any pre-conception on behalf of the investigators of populations that would be changed by experimental conditions. CITRUS was effective for tracking populations across time and the sensitivity inherent in relatively high sub-division of the populations facilitated early detection of the impact of culturing cells in the presence of iDL1.

## Conclusions

The Notch pathway can be used in a clinically compliant manner to generate a safe and potentially efficacious cell therapy product [23]. The approach discussed has the potential to improve the controlled scalability (in particular volume to surface area) and cell fate control of ex vivo expansion of HSCs for clinical use. It further has the potential for application with other protein targets important in hematopoietic cell expansion such as Jagged 1/2, Wnt ligands and integrins [43]. In this regard the methodology potentially provides an opportunity to recapitulate some wider surface presented aspects of the stem cell niche and control a wider range of biological outcomes in simple scalable systems.

## Additional files

Additional file 1: Characterisation of starting cell population. Flow cytometry histograms for CD33, CD34, CD38, CD45RA, CD133 and CD135 of CD34^+^ enriched cells at day 0 and day 6. (PPTX 3620 kb)

Additional file 2: Live cell culture images. Characterisation of cell and particle interaction and aggregation during culture at 40× and 20× magnification. (DOCX 1013 kb)

## Abbreviations

BM: Bone marrow
CITRUS: Cluster identification, characterisation and regression
DL1: Delta-like 1
ELISA: Enzyme-linked immunosorbent assay
fg: Femtogram
FLT3: Flt3/flk2 ligand
GVHD: Graft versus host disease
HSC: Hematopoietic stem cell
iDL1: Immobilised Delta-like 1
IL-3: Interleukin-3
MPB: Mobilised peripheral blood
NHS: N-Hydroxysuccinimide
PEG: Polyethylene glycol
SCF: Stem cell factor
TPO: Thrombopoietin
UCB: Umbilical cord blood
