# Supplementary figures and images for: Immobilisation of Delta-like 1 ligand for the scalable and controlled manufacture of hematopoietic progenitor cells in a stirred bioreactor
Source: BMC Biotechnol. 2017 Aug 4;17:65. doi: 10.1186/s12896-017-0383-0 (PMC5544980; doi:10.1186/s12896-017-0383-0)

## Slide 1
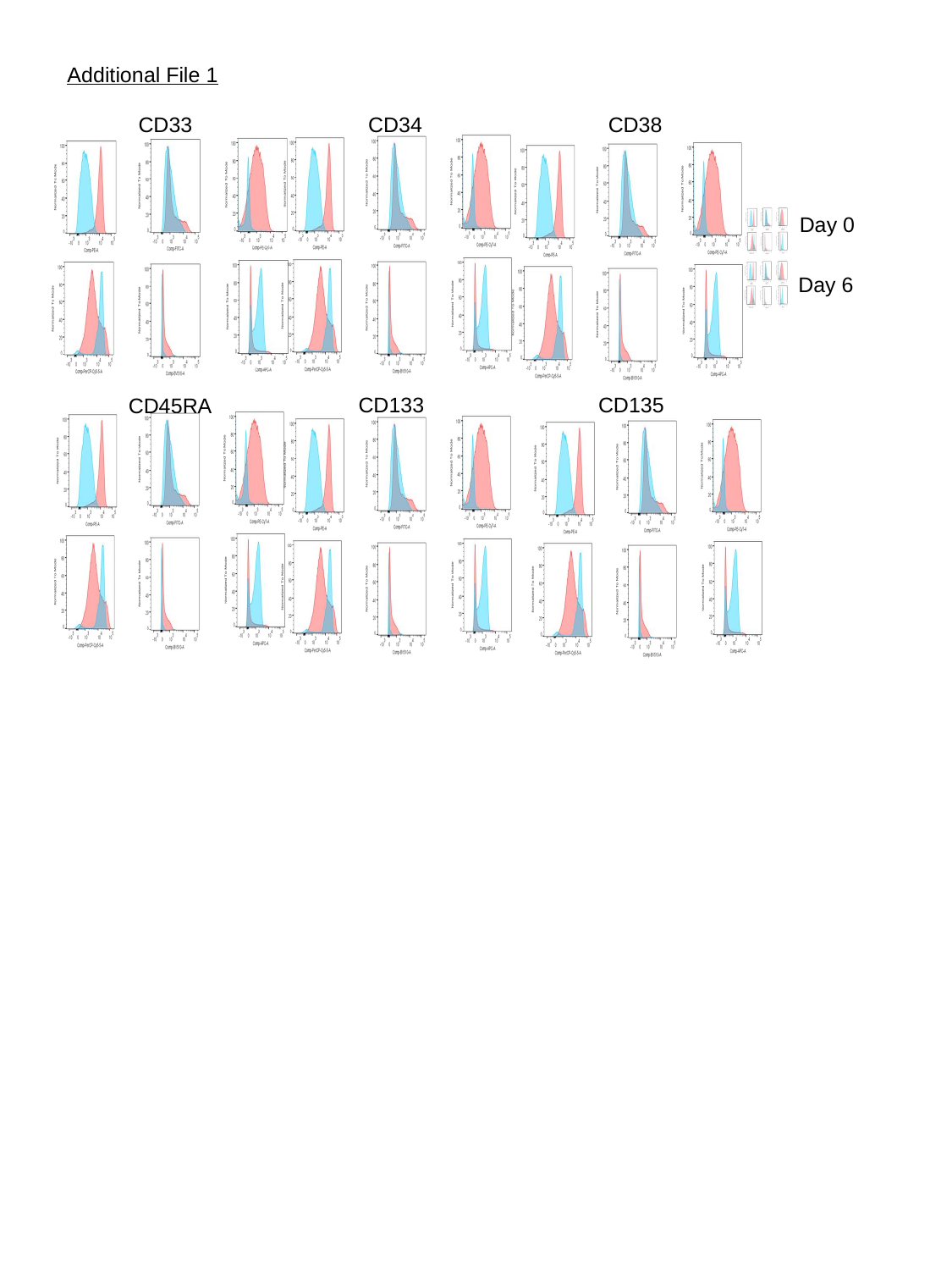

Additional File 1
CD33
CD34
CD38
CD133
CD135
CD45RA
Day 0
Day 6

Supplement: Supplementary file 1 — Characterisation of starting cell population. Flow cytometry histograms for CD33, CD34, CD38, CD45RA, CD133 and CD135 of CD34+ enriched cells at day 0 and day 6. (PPTX 3620 kb) [file 12896_2017_383_MOESM1_ESM.pptx]

Additional File 2


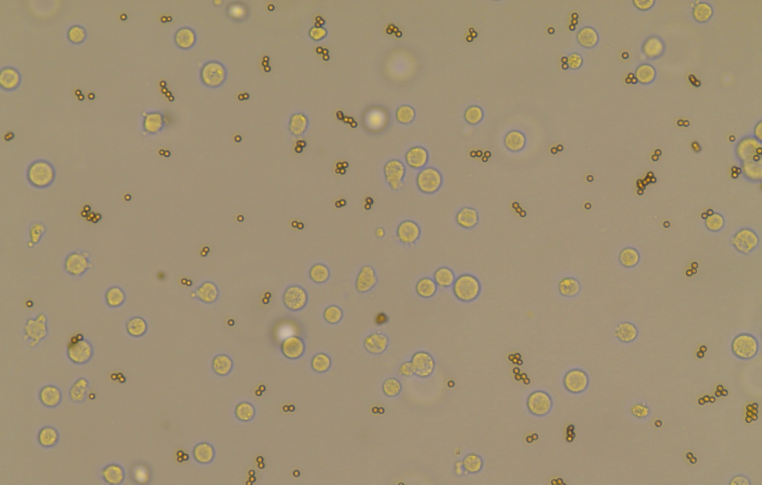

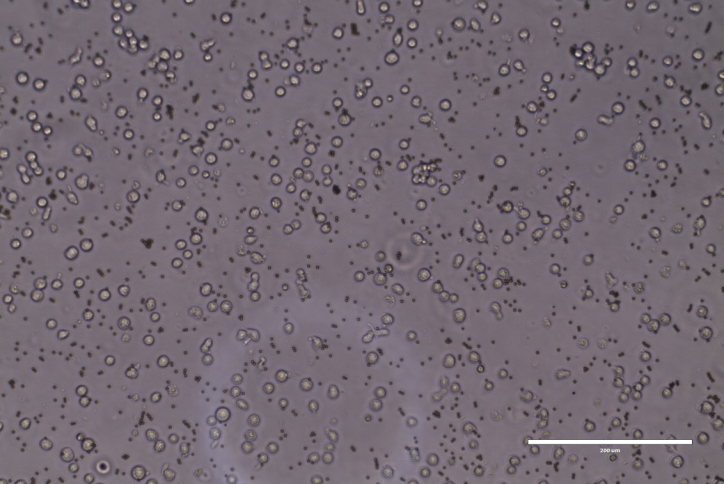

Supplement: Supplementary file 2 — Live cell culture images. Characterisation of cell and particle interaction and aggregation during culture at 40× and 20× magnification. (DOCX 1013 kb) [file 12896_2017_383_MOESM2_ESM.docx]
